# Spontaneous showering of tumor emboli in a patient with advanced primary lung cancer: a case report

**DOI:** 10.1186/1865-1380-5-27

**Published:** 2012-06-06

**Authors:** Susan M Schreffler, William F Paolo, Brian T Kloss

**Affiliations:** 1 SUNY Upstate Medical University, Department of Emergency Medicine, 550 E. Genesee Street, Syracuse, NY, USA

## Abstract

Extension of primary lung tumors into the left atrium via pulmonary veins is a well-documented phenomenon. Peripheral arterial embolism and cerebral embolism originating from a primary lung neoplasm are rare events. We report a case of simultaneous acute bilateral lower limb ischemia, bilateral renal infarction, splenic infarction and cerebral infarction as a result of multiple emboli originating from primary lung malignancy invasion of the left atrium. An emergent embolectomy revealed pathologic features of the extracted thrombus that were identical to the pulmonary neoplasm.

## Background

Primary pulmonary malignancy with left atrial extension via the pulmonary veins has been well documented [1-3]. Neoplasms involving the pulmonary veins and atria can induce spontaneous systemic emboli and circulatory compromise leading to acute severe complications [4]. Primary or metastatic lung tumors are reported to be the most common source of cardiogenic emboli of neoplastic origin [5]. Peripheral arterial embolization of neoplastic origin is rare. When it does occur, the most common embolic source is a malignant primary or metastatic pulmonary tumor that erodes through the wall of a pulmonary vein and embolizes through fragmentation [6]. The following case report describes a 62-year-old patient with a recently diagnosed advanced primary lung cancer who presented to the emergency department with a resultant showering of tumor emboli to multiple arterial sites.

## Case presentation

A 62-year-old male with a history of hypertension, COPD, GERD and recently diagnosed poorly differentiated adenocarcinoma of the left lung with invasion of his left pulmonary vein and left atrium presented to the emergency department (ED) with intermittent aphasia, complaining of left flank pain and bilateral lower extremity pain and numbness. The patient was on anticoagulation therapy and scheduled to have combined cardiothoracic surgery for resection of the left lung and left atrial masses.

On initial evaluation, he was in obvious distress, writhing in bed, with a blood pressure of 141/52 mmHg, a heart rate of 60/min, a respiratory rate of 16/min, a peripheral O_2_ saturation of 99% on room air, and a temperature of 37.4 °C. He exhibited intermittent fluent aphasia. Cranial nerves II - XII were intact. Upper extremity examination revealed normal circulation, motor and sensation bilaterally. Lower extremities were pallorous and cold, the left greater than the right. There were palpable bilateral femoral pulses, nonpalpable bilateral popliteal pulses, and nonpalpable and non-dopplerable bilateral dorsalis pedis, and posterior tibial pulses. Strength was noted to be 0/5 on the right and 2/5 on the left. Sensation was decreased to light touch and pinprick, with the left greater than the right. Deep tendon reflexes were symmetrical.

**Figure 1:**
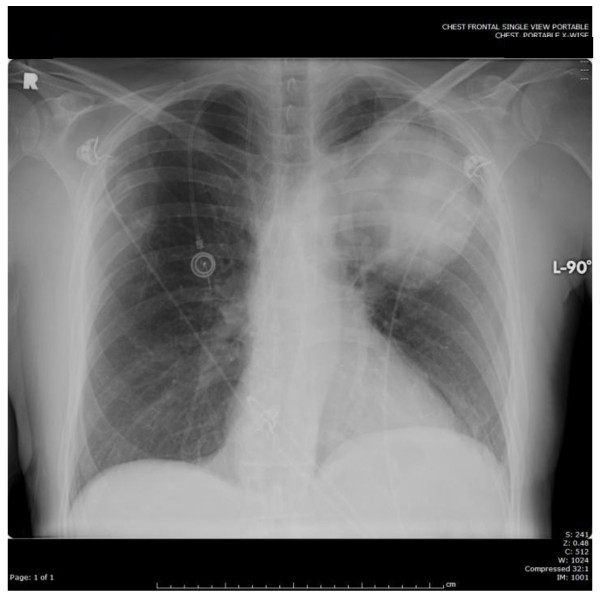
Por Chest X-ray showing the left upper lobe lung mass.

**Figure 2:**
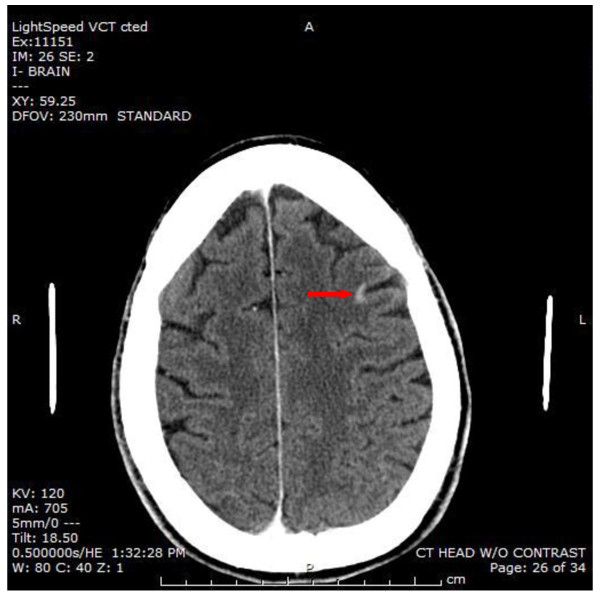
**Computerized tomography demonstrating acute cerebral infarction in the left frontal lobe (*arrow*).**

**Figure 3:**
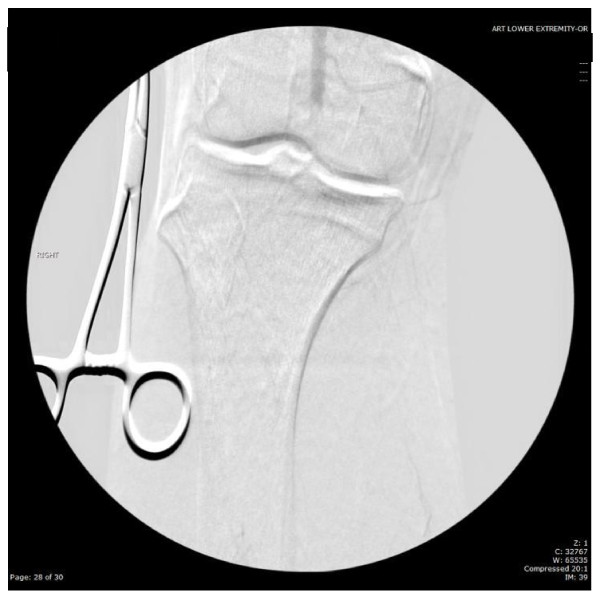
Computerized tomography angiogram demonstrating right popliteal artery occlusion.

**Figure 4:**
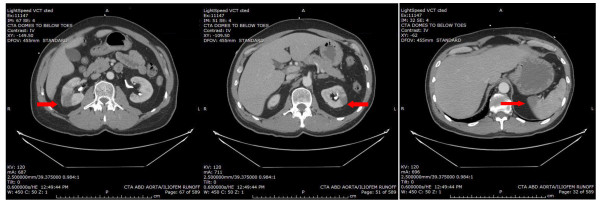
**Computerized tomography shows bilateral renal infarctions and spleen infarction (*arrows*).**

His white blood cell count (16,700/μl) was mildly elevated, and his PTT (41.7 s) was prolonged. An EKG showed a sinus rhythm with a right bundle branch block that was unchanged from a prior EKG. A por chest X-ray (Figure 1) showed a large left upper lobe mass. A brain CT demonstrated a small subacute cortical infarct in the left frontal lobe (Figure 2). A CT angiogram of the aorta w ith runoff revealed occlusion of the left external iliac artery without collateral or distal reperfusion, and occlusion of the right popliteal artery (Figure 3) without collateral or distal reperfusion. It also showed bilateral renal infarcts and a splenic infarct (Figure 4).

Since the patient was on Lovenox, he was not heparinized in the emergency department. He was evaluated by neurology and vascular surgery, and emergently taken to the operating room for bilateral thromboembolectomies and bilateral four-compartment fasciotomies. Improvement in lower extremity circulation was immediate upon re-establishment of blood flow with return of distal pulses. A histological examination of the emboli confirmed metastatic carcinoma consistent with poorly differentiated adenocarcinoma.

Postoperatively, given the patient’s condition on admission, he was no longer deemed a surgical candidate for mass excision. Hematology/oncology and radiation oncology were consulted, and they suggested palliative chemotherapy and radiation after his fasciotomy incisions healed. On postoperative day 3, the patient developed atrial fibrillation and was placed on diltiazem. On postoperative day 8, he was discharged home.

## Discussion

Venous thrombosis is a frequent complication of malignancy; however, acute arterial occlusion secondary to malignant (non-myxomatous) tumor embolism is a rare event [7]. Venous tumor emboli most often present with symptoms of pulmonary embolism and/or infarction [8]. Arterial embolism results in organ ischemia/infarction, and must be recognized and managed appropriately [6]. In most cases, a primary or metastatic pulmonary neoplasm gains access to the arterial system by invading the heart through the pulmonary veins [5]. Fewer than ten cases of spontaneous tumor embolization resulting from lung cancer invasion of the pulmonary vein have been reported [5]. The sites of tumor emboli reported most frequently are the aortic bifurcation or femoral vessels (50%), and the cerebral circulation (30%) [9]. Patient symptoms are related to the embolic location, and most commonly include lower extremity, cerebral, myocardial, and limb ischemic events [10]. To our knowledge, this is the first reported case of simultaneous non-myxomatous tumor embolization to the brain, spleen, kidneys and bilateral lower extremities.

Cerebral ischemia has several major etiologies, including atherosclerosis, cardiogenic emboli, vasculitis, increased blood viscosity and carotid dissection [11]. About 80% of all cerebrovascular events are ischemic in origin, and most are associated with atherosclerotic disease [11]. Cardiogenic emboli account for 15–30% of ischemic strokes [5]. Embolic strokes are characteristically abrupt in onset.

Acute limb ischemia is typically categorized as thrombotic, embolic or traumatic [12], and characteristically described by the six Ps: pain, pallor, pulselessness, paresthesias, poikilothermia and paralysis [13]. Embolization of the peripheral artery is most commonly cardiogenic (80–90%), but emboli originating from a malignant tumor are rare [4].

The vast majority (88%) of splenic infarctions are caused by either infiltrative hematologic diseases resulting in congestion of the splenic circulation by abnormal cells or thromboembolic conditions causing vessel obstruction [14]. In a 10-year retrospective study, Antopolsky et al. examined clinical presentations in 49 episodes of acute splenic infarction. The most common symptom was either abdominal or left flank pain (80% of episodes), while the most common sign was upper left quadrant tenderness (35% of episodes) [15].

There are two major causes of acute renal infarction: thromboemboli, usually originating from a thrombus in the left atrium or aorta, and less commonly, a thrombosis within a renal artery [16]. Other rare potential embolic sources include valvular vegetations, tumor and fat emboli, and paradoxical embolism through a patent foramen ovale [17]. Because patients present with abdominal or flank pain that mimics other conditions, such as nephrolithiasis and pyelonephritis, renal infarction is under-diagnosed and frequently missed [18]. The patient described in our study presented simultaneously with an acute onset of intermittent aphasia suggestive of an acute cerebral embolic event, bilateral lower extremity pain and paresthesias suggestive of acute limb ischemia, and left flank pain suggestive of splenic and/or renal infarction.

Arteriography is the diagnostic procedure of choice for identifying an acute arterial occlusion. In addition to demonstrating detailed arterial anatomy, arteriography can usually distinguish between thrombosis and embolism [19]. An embolus will often demonstrate a sharp cutoff with a rounded reverse meniscus sign. The embolus may also be visible as an intraluminal filling defect if the vessel is not completely occluded. Other findings that are most consistent with an embolus include the presence of otherwise normal vessels, the absence of collateral circulation and the presence of multiple filling defects. Arterial thrombosis is usually visualized as a sharp or tapered, but not rounded cutoff on arteriography. Diffuse atherosclerosis with well-developed collateral circulation is generally present [20].

Once an embolism has been identified, an embolectomy should be emergently performed, and anticoagulation and vasodilators started [7]. In our case, the patient also required bilateral fasciotomies to treat revascularization compartment syndrome.

## Conclusions

This case was a rare and extreme example of the potential sequelae in a patient with advanced primary lung cancer that has invasion of a pulmonary vein with extension into the left atria. Although tumor emboli to the cerebral and peripheral circulation are rare, the possibility should be considered in the differential diagnosis of strokes and arterial emboli in a patient with lung malignancies.

## Consent

Written informed consent was obtained from the patient for publication of this report and any accompanying images.

## Competing interests

The authors declare that they have no competing interests.

## Authors’ contributions

SS obtained photographs, prepared images, reviewed reports and performed literature searches. WP participated in the care of the patient and provided case details. All authors reviewed the literature and provided authorship of the text of this manuscript. All authors read and approved the final manuscript.
